# Do we have scientific evidence about the effect of hypoxaemia on cognitive outcome in adult patients with severe acute respiratory failure?

**DOI:** 10.1080/03009734.2018.1433255

**Published:** 2018-02-27

**Authors:** Bernhard Holzgraefe, Anders Larsson, Laura Von Kobyletzki

**Affiliations:** Department of Physiology and Pharmacology, Karolinska Institutet, Stockholm, Sweden; Department of Anaesthesia, Surgical Services and Intensive Care Medicine, County Council of Värmland, Arvika Community Hospital, Arvika, Sweden; Anaesthesiology and Intensive Care, Department of Surgical Sciences, Hedenstierna Laboratory, Uppsala University, Uppsala, Sweden; Department of Dermatology, Lund University, Skåne University Hospital, Malmö, Sweden; Department of Public Health Sciences, Karlstad University, Karlstad, Sweden

Dear Editor,

Survivors of acute respiratory failure (ARF) treated with invasive mechanical ventilation (IMV) only or IMV in combination with extracorporeal membrane oxygenation (ECMO) can suffer from mental disorders later on, especially from cognitive impairment (1). It was suggested that long-term cognitive impairment could be caused by hypoxaemia, which is a common condition during the course of severe ARF in adult patients (2,3). To shed some light on this matter we performed a systematic literature search. We included cohort studies, nested case-control studies, or randomized controlled trials that evaluated associations between hypoxaemia and cognitive function and the effect of ECMO during the course of ARF on cognitive functioning. Hypoxaemia was defined as a haemoglobin oxygen saturation (SaO_2_) < 94%. Only studies performed in adult patients who were treated with IMV with or without ECMO were included. Study eligibility was independently assessed by two reviewers (authors B.H., L.K.) as were primary selected studies extracted using a standardized form.Full details on our methodology have been included in our protocol registration on the International Prospective Register of Systematic Reviews (PROSPERO 2016: CRD42016045447) and as supplemental materials (Search strategy, available online). The search in Medline, EMBASE, Cochrane, and PsycInfo was finalized on 26 August 2016.

A total of 2606 articles were found. After removal of 781 duplicates, 1825 titles and abstracts were screened (Preferred Reporting Items for Systematic Reviews and Meta-Analyses, PRISMA 2009; Figure 1) (4). Thirty papers were identified for full-text assessment. We could not identify any study that met the inclusion criteria. However, four case series and two longitudinal studies without comparison to an unexposed control group investigating study questions similar to the current study were found (see Search Strategy, available online).

**Figure 1.**
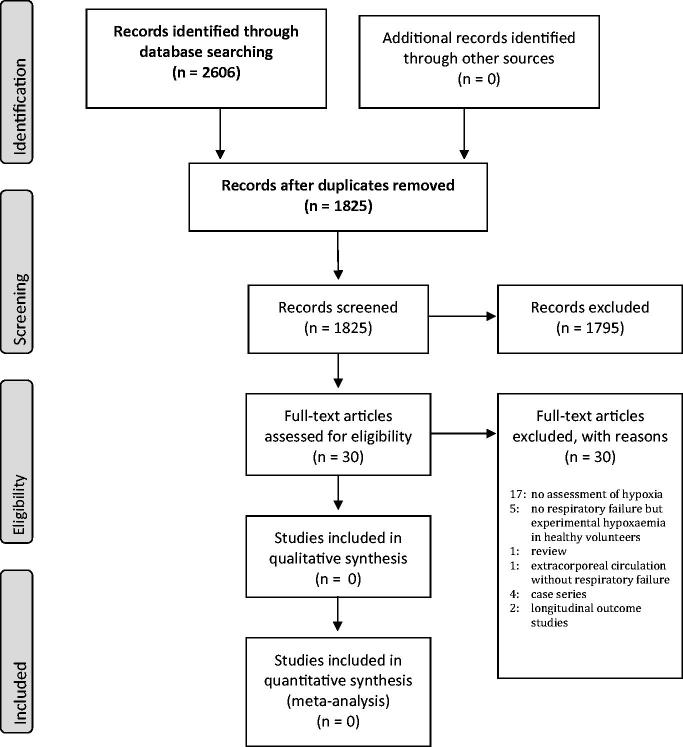
PRISMA 2009 flow diagram.

A seventh case series, which was published online in October 2016, was added after the database search (Supplementary Tables 1–3, available online). Two studies showed some evidence that arterial oxygen saturation below 90% might be associated with cognitive impairment (studies 22 and 24, Supplementary Tables 1–3, available online). One study found a correlation between the duration of desaturation and cognitive outcome at 1-year follow-up and another study at discharge but not at 1- and 2-year follow-up. The patients in these studies were mechanically ventilated and without ECMO treatment. One study including patients with ECMO treatment did not find an association between hypoxaemia and cognitive impairment (study 25, Supplementary Tables 1–3, available online).

Our search highlights a dearth of evidence on the association of hypoxaemia during the course of ARF with or without ECMO and cognitive function, with not a single study fulfilling our eligibility criteria. The case series found conflicting results. This was in line with the first International Study of Postoperative Cognitive Dysfunction, which did not find any association between hypoxaemia and cognitive dysfunction (5).

The occurrence of hypoxaemia is a common feature in severe ARF and other conditions with severely impaired pulmonary gas exchange. Hypoxaemia is defined as haemoglobin oxygen saturation below the normal value. The term ‘normoxaemia’ is defined as a PaO_2_ of 10.7–13.3 kPa (80–100 mmHg) or a SaO_2_ of >94% at sea level (6). On the other hand, the term hypoxia describes a lack of oxygen at the cellular level. The terms hypoxaemia and hypoxia are often mixed up, but it is important to keep in mind that they describe two different situations. Hypoxaemia may not lead to cellular hypoxia per se because the physiological compensation of reductions in oxygen saturation might maintain capillary oxygen content at an adequate level (7), which seems to be the most important factor for sufficient cerebral oxygenation (8). Tissue hypoxia is usually caused by an ischemic event, i.e. cerebral infarction. Therefore, in our view, it is unlikely that hypoxaemia per se will lead to tissue hypoxia as long as organ perfusion is preserved. Attempts to maintain normoxaemia during treatment with mechanical ventilation with or without ECMO in patients with hypoxaemic respiratory failure are associated with a risk of serious complications, e.g. ventilator-induced lung injury (9) and cerebral haemorrhage (10). This risk could even out the potential benefit of increasing blood oxygen saturation. Thus, it is important to clarify whether hypoxaemia is associated with decreased cognitive function.

Our observations highlight a knowledge gap on this issue. From low-evidence data we cannot exclude that hypoxaemia could negatively influence cognitive function at discharge from hospital in patients treated with only mechanical ventilation. Hence, we conclude that future high-quality studies are needed to explore this question.

## Supplementary Material

Supplemental data
