# Supplementary material for: Do we have scientific evidence about the effect of hypoxaemia on cognitive outcome in adult patients with severe acute respiratory failure?
Source: Ups J Med Sci. 2018 Feb 27;123(1):68–70. doi: 10.1080/03009734.2018.1433255 (PMC5901471; doi:10.1080/03009734.2018.1433255)
Supplement: Supplemental data [file IUPS_A_1433255_SM2341.zip › IUPS_Supp_mat/Appendix Search strategy .docx]

Appendix 1 Search strategy Medline

| Date of Search: 26 Aug. 2016  Number of hits: 843  Comments: | Field labels:   - exp = MeSH - ti,ab = title & abstract - kf = keyword - adj3 = within three words |
| --- | --- |
| 1. exp Respiratory Distress Syndrome, Adult/ 2. (ards).ti,ab,kf.  3. ((respirator*) adj3 (distress or fail*)).ti,ab,kf. 4. exp Extracorporeal Membrane Oxygenation/ 5. ((extracorporeal OR extra corporeal OR extrapulmonar* OR extra pulmonar*) adj3 (membrane OR oxygen* OR pump OR lung assist* OR life support*)).ti,ab,kf.  6. ^38^.ti,ab,kf. 7. exp Hypoxia, Brain/ 8. (anoxaemia OR anoxemia OR anoxia OR asphyxia OR hypoxia OR hypoxemia).ti,ab,kf.  9. exp Anoxia/ 10. (oxygen adj3 lack).ti,ab,kf. 11. exp Acute Lung Injury/ 12. acute lung injur*.ti,ab,kf.  13. 1-12/OR  14. exp Cognition Disorders/ 15. exp Memory Disorders/ 16. (cognition OR cognitive OR neurocogn* OR memory).ti,ab,kf.  17. exp Stress Disorders, Post-Traumatic/ 18. ((post-traumatic OR posttraumatic OR post traumatic) adj3 (stress OR neuros* OR psychos* OR disorder*)).ti,ab,kf.  19. ptsd.ti,ab,kf.  20. exp Quality of Life/ 21. (quality of life OR HRQL OR life quality).ti,ab,kf.  22. exp Depression/ OR exp Depressive Disorder/ 23. depress*.ti,ab,kf.  24. exp Brain Damage, Chronic/  25. ((chronic) adj3 (brain damage OR brain lesion OR damage of the brain OR encephalopath*)).ti,ab,kf. 26. 14-25/OR  27. exp Psychological Tests/ 28. (WAIS* OR Wechsler adult intelligence scale OR Halstead Reitan neuropsychological battery OR luria Nebraska neuropsychological battery OR mini mental state examination OR task switching OR Wechsler memory scale OR Benton revised visual retention test OR Kohs block design test OR Miller analogies test OR Peabody picture vocabulary test OR porteus maze test OR Wechsler bellevue intelligence scale OR Stanford binet intelligence scale OR Slosson intelligence test OR raven progressive matrices).ti,ab,kf. 29. ((intelligen* OR psycho* OR neuro* OR cognit* OR aptitude OR Bender-gestalt OR stroop OR trail making) adj3 (test* OR measure* OR assess* OR examinat* OR questionnaire* OR battery OR perform* OR deficit*)).ti,ab, kf. 30. 27-29/OR 31. 13 AND 26 AND 30 32. exp animals/ not exp humans/ 33. 31 NOT 32 34. limit 33 to ("all infant (birth to 23 months)" or "all child (0 to 18 years)" or "newborn infant (birth to 1 month)" or "infant (1 to 23 months)" or "preschool child (2 to 5 years)" or "child (6 to 12 years)" or "adolescent (13 to 18 years)") 35. limit 33 to ("all adult (19 plus years)" or "young adult (19 to 24 years)" or "adult (19 to 44 years)" or "young adult and adult (19-24 and 19-44)" or "middle age (45 to 64 years)" or "middle aged (45 plus years)" or "all aged (65 and over)" or "aged (80 and over)")  36. 34 NOT 35  37. 33 NOT 36 | |

Appendix 2 Search strategy Psycinfo

| Date of Search: 2016-08-26  Number of hits: 526  Comments: | Field labels:   - Same as Medline above |
| --- | --- |
| 1. Respiratory Distress 2. (ards).ti,ab. 3. (respirator* adj3 (distress or fail*)).ti,ab. 4. exp Oxygenation/ 5. ((extracorporeal OR extra corporeal OR extrapulmonar* OR extra pulmonar*) adj3 (membrane OR oxygen* OR pump OR lung assist* OR life support*)).ti,ab. 6. ^38^.ti,ab. 7. exp Anoxia / 8. (anoxaemia OR anoxemia OR anoxia OR asphyxia OR hypoxia OR hypoxemia).ti,ab. 9. (oxygen adj3 lack).ti,ab. 10. exp Lung Disorders/ 11. acute lung injur*.ti,ab. 12. 1-12/OR 13. exp Cognitive Ability/ OR exp Cognitive Impairment/ 14. exp Memory Disorders/ 15. (cognition OR cognitive OR neurocogn* OR memory).ti,ab. 16. exp Posttraumatic Stress Disorder/ 17. ((post-traumatic OR posttraumatic OR post traumatic) adj3 (stress OR neuros* OR psychos* OR disorder*)).ti,ab. 18. ptsd.ti,ab. 19. exp Quality of Life/ 20. (quality of life OR HRQL OR life quality).ti,ab. 21. exp Depression (emotion)/ 22. exp Major Depression/ 23. depress*.ti,ab. 24. exp Brain Damage/ 25. ((chronic) adj3 (brain damage OR brain lesion OR damage of the brain OR encephalopath*)).ti,ab. 26. 13-25/OR 27. exp Intelligence Measures/ 28. exp Neuropsychological Assessment/ 29. ((WAIS* OR Wechsler adult intelligence scale OR Halstead reitan neuropsychological battery OR luria Nebraska neuropsychological battery OR mini mental state examination OR task switching OR Wechsler memory scale OR Benton revised visual retention test OR Kohs block design test OR Miller analogies test OR Peabody picture vocabulary test OR porteus maze test OR Wechsler bellevue intelligence scale OR Stanford binet intelligence scale OR Slosson intelligence test OR raven progressive matrices).ti,ab. 30. ((intelligen* OR psycho* OR neuro* OR cognit* OR aptitude OR Bender-gestalt OR stroop OR trail making) adj3 (test* OR measure* OR assess* OR examinat* OR questionnaire* OR battery OR perform* OR deficit*)).ti,ab. 31. 27-30/OR 32. 12 AND 26 AND 31 33. limit 32 to (100 childhood <birth to age 12 yrs> or 120 neonatal <birth to age 1 mo> or 140 infancy <2 to 23 mo> or 160 preschool age <age 2 to 5 yrs> or 180 school age <age 6 to 12 yrs> or 200 adolescence <age 13 to 17 yrs>) 34. limit 32 to ("300 adulthood <age 18 yrs and older>" or 320 young adulthood <age 18 to 29 yrs> or 340 thirties <age 30 to 39 yrs> or 360 middle age <age 40 to 64 yrs> or "380 aged <age 65 yrs and older>" or "390 very old <age 85 yrs and older>") 35. 33 not 34 36. 32 not 35 | |

Appendix 3 Search strategy Cochrane Library (Wiley)

| Date of Search: 2016-08-24  Number of hits: 147   - Cochrane Reviews (13) - Other Reviews (1) - Trials (132) | Field labels: ti,ab,kw = title, abstract, keywords |
| --- | --- |
| 1. (ards OR ecmo OR anoxaemia OR anoxemia OR anoxia OR asphyxia OR hypoxia OR hypoxemia OR acute lung injur*):ti,ab,kw 2. ((respirator*) NEAR/3 (distress or fail*)): ti,ab,kw 3. ((extracorporeal OR extra corporeal OR extrapulmonar* OR extra pulmonar*) NEAR/3 (membrane OR oxygen* OR pump OR lung assist* OR life support*)): ti,ab,kw 4. (oxygen NEAR/3 lack): ti,ab,kw 5. #1 or #2 or #3 OR #4 6. (cognition OR cognitive OR neurocogn* OR memory):ti,ab,kw 7. ((post-traumatic OR posttraumatic OR post traumatic) NEAR/3 (stress OR neuros* OR psychos* OR disorder*)):ti,ab,kw 8. PTSD:ti,ab,kw 9. (quality of life OR hrql OR life quality):ti,ab,kw 10. depress*:ti,ab,kw 11. ((chronic) NEAR/3 (brain damage OR brain lesion OR damage of the brain OR encephalopath*)):ti,ab,kw 12. #6 or #7 or #8 or #9 or #10 or #11 13. ((WAIS* OR Wechsler adult intelligence scale OR Halstead reitan neuropsychological battery OR luria Nebraska neuropsychological battery OR mini mental state examination OR task switching OR Wechsler memory scale OR Benton revised visual retention test OR Kohs block design test OR Miller analogies test OR peabody picture vocabulary test OR porteus maze test OR Wechsler bellevue intelligence scale OR Stanford binet intelligence scale OR Slosson intelligence test OR raven progressive matrices):ti,ab,kw 14. ((intelligen* OR psycho* OR neuro* OR cognit* OR aptitude OR Bender-gestalt OR stroop OR trail making) NEAR/3 (test* OR measure* OR assess* OR examinat* OR questionnaire* OR battery OR perform* OR deficit*)):ti,ab,kw 15. #13 or #14 16. 5 AND 12 AND 15 | |

Appendix 4 Search strategy EMBASE (embase.com)

| Date of Search: 2016-08-26  Number of hits: 1,091  Comments: | Field labels:   - ab,ti = abstract & title - NEAR/3 = within three words - exp = Emtree |
| --- | --- |
| **No.**  **Query**  **Results**  **1,091**  **#37**  **#35** NOT **#36**  **2,233,709**  **#36**  [adolescent]/lim OR [child]/lim OR [embryo]/lim OR [fetus]/lim OR [infant]/lim OR [newborn]/lim OR [preschool]/lim OR [school]/lim NOT ([adult]/lim OR [aged]/lim OR [middle aged]/lim OR [very elderly]/lim OR [young adult]/lim)  **526**  **#35**  **#33** NOT **#34**  **5,008,119**  **#34**  [animals]/lim NOT [humans]/lim  **546**  **#33**  **#14** AND **#28** AND **#32**  **252,083**  **#32**  **#29** OR **#30** OR **#31**  **308,055**  **#31**  ((**intelligen*** OR **psycho*** OR **neuro*** OR **cognit*** OR **aptitude** OR **'bender-gestalt'** OR **stroop** OR **'trail making'**) NEAR/3 (**test*** OR**measure*** OR **assess*** OR **examinat*** OR **questionnaire*** OR **battery** OR **perform*** OR **deficit***)):ab,ti  **66,808**  **#30**  **WAIS***:ab,ti OR **'Wechsler adult intelligence scale'**:ab,ti OR **'Halstead reitan neuropsychological battery'**:ab,ti OR **'luria Nebraska neuropsychological battery'**:ab,ti OR **'mini mental state examination'**:ab,ti OR **'task switching'**:ab,ti OR **'Wechsler memory scale'**:ab,ti OR **'Benton revised visual retention test'**:ab,ti OR **'Kohs block design test'**:ab,ti OR **'Miller analogies test'**:ab,ti OR **'peabody picture vocabulary test'**:ab,ti OR **'porteus maze test'**:ab,ti OR **'Wechsler bellevue intelligence scale'**:ab,ti OR **'Stanford binet intelligence scale'**:ab,ti OR **'Slosson intelligence test'**:ab,ti OR **'raven progressive matrices'**:ab,ti  **178,618**  **#29**  **'psychologic test'**/exp  **1,284,858**  **#28**  **#15** OR **#16** OR **#17** OR **#18** OR **#19** OR **#20** OR **#21** OR **#22** OR **#23** OR **#24** OR **#25** OR **#26** OR **#27**  **1,763**  **#27**  (**chronic** NEAR/3 (**'brain damage'** OR **'brain lesion'** OR **'damage of the brain'** OR **encephalopathy**)):ab,ti  **5,721**  **#26**  **'chronic brain disease'**/exp  **474,738**  **#25**  **depress***:ab,ti  **374,571**  **#24**  **'depression'**/exp  **279,347**  **#23**  **'quality of life'**:ab,ti OR **hrql**:ab,ti OR **'life quality'**:ab,ti  **345,901**  **#22**  **'quality of life'**/exp  **20,490**  **#21**  **ptsd**:ab,ti  **27,740**  **#20**  ((**'post-traumatic'** OR **posttraumatic** OR **'post traumatic'**) NEAR/3 (**stress** OR **neuros*** OR **psychos*** OR **disorder***)):ab,ti  **43,374**  **#19**  **'posttraumatic stress disorder'**/exp  **527,868**  **#18**  **cognition**:ab,ti OR **cognitive**:ab,ti OR **neurocogn*** OR **memory**:ab,ti  **60,607**  **#17**  **'memory disorder'**/exp  **15,048**  **#16**  **'mild cognitive impairment'**/exp  **120,395**  **#15**  **'cognitive defect'**/exp  **311,227**  **#14**  **#1** OR **#2** OR **#3** OR **#4** OR **#5** OR **#6** OR **#7** OR **#8** OR **#9** OR **#10** OR **#11** OR **#12** OR **#13**  **14,603**  **#13**  **'acute lung injur*'**:ab,ti  **10,878**  **#12**  **'acute lung injury'**/exp  **938**  **#11**  (**oxygen** NEAR/3 **lack**):ab,ti  **150,970**  **#10**  **anoxaemia**:ab,ti OR **anoxemia**:ab,ti OR **anoxia**:ab,ti OR **asphyxia**:ab,ti OR **hypoxia**:ab,ti OR **hypoxemia**:ab,ti  **138,049**  **#9**  **'hypoxemia'**/exp  **10,608**  **#8**  **'brain hypoxia'**/exp  **8,055**  **#7**  **ecmo**:ab,ti  **11,167**  **#6**  ((**extracorporeal** OR **'extra corporeal'** OR **extrapulmonar*** OR **'extra pulmonar*'**) NEAR/3 (**membrane** OR **oxygen*** OR **pump** OR **'lung assist*'** OR **'life support*'**)):ab,ti  **527**  **#5**  **'extracorporeal membrane oxygenation device'**/exp  **13,272**  **#4**  **'extracorporeal oxygenation'**/exp  **77,949**  **#3**  (**respirator*** NEAR/3 (**distress** OR **fail***)):ab,ti  **13,790**  **#2**  **ards**:ab,ti  **26,632**  **#1**  **'adult respiratory distress syndrome'**/exp | |
