# Supplementary material for: Do we have scientific evidence about the effect of hypoxaemia on cognitive outcome in adult patients with severe acute respiratory failure?
Source: Ups J Med Sci. 2018 Feb 27;123(1):68–70. doi: 10.1080/03009734.2018.1433255 (PMC5901471; doi:10.1080/03009734.2018.1433255)
Supplement: Supplemental data [file IUPS_A_1433255_SM2341.zip › IUPS_Supp_mat/Table 1_2_3_ UJMS.docx]

Table 1 Characteristics of the studies

| Author, year of publication | Study design | Control group | Main exposure | Study size | Mean age, years | Follow up time | Respiratory failure/ARDS stage | Exclusion |
| --- | --- | --- | --- | --- | --- | --- | --- | --- |
| Hopkins RO^22^, 1999 | Case series | Normative population mean | Duration of desaturation  with SaO2 <90%  <85% and <80% | enrolled: n = 106 study completed at 1 year follow up: n=55 | 45.5 (16 – 78) | Dc and 1 year | All/unclear | Irreversible disease states, 1  year survival unlikely, enrolled in another study,  immunosuppression, CNS damage, inability to obtain informed consent, severe ARDS > 21 days, malignancy, primary care physician refused consent, chronic renal failure, pregnancy, pneumonectomy, chronic heart failure |
| Rothenhäusler H-B^13^, 2001 | Case series | Normative population mean | ARDS: n = 40 ECMO: N = 6 | evaluated: n = 119  included: n = 46 | 41.5 (+/- 14.7) | Median 6 years (range 1-12 years) | All/unclear | none |
| Hopkins RO^23^, 2004 | Prospective longitudinal  outcome study | None | ARDS patients from a ventilation trial; duration SaO_2_ <90% | evaluated: n = 78  completed dc  n = 74  completed 1 year  follow-up: n = 66 | 45.8 (16-81) | 1year | All/P/F ratio ≤150 mmHg | Premorbid cognitive disability, traumatic brain injury, neurologic disease, psychotic  disorder |
| Hopkins RO^24^, 2005 | Longitudinal outcome | Premorbid IQ estimation with OPIE of the study participants | Duration of desaturation  SaO_2_<90% | evaluated: n = 120, included: n = 74 | 46 (35-57) | 2 years | All/unclear | 2 with cognitive disability, 1with Alzheimer’s disease |
| Risnes I^12^, 2006 | Case series | None | n = 28,  v-v: n = 12  v-a: n = 16 | n = 28 | 37.9 (18.8 - 63.5) | Mean 5 years (0.5 – 12 years) | 11/unclear | age <18 years |
| Mikkelsen ME^10^, 2012 | Prospective case series | Normative population mean | Hypoxaemia, no definition | consented: n = 213 tested: n = 102 completed all domains : n = 75 | 49 (40-58) | 12 month | All/unclear | None |
| Holzgraefe B^25^, 2016 | Retrospective  case series | Normative population mean | SaO_2_ ±94% during first 10 days or whole treatment if shorter than 10 days | evaluated 11,  included 7 | 34.7 (±12) | 3.2 years | All/severe ARDS | none |

ECMO: extracorporeal membrane oxygenation; ARDS: acute respiratory distress syndrome; P/F ratio: ratio of arterial oxygen partial pressure to fractional inspired oxygen; v-a ECMO: veno-arterial ECMO; v-v ECMO: veno-venous ECMO; dc: discharge; SaO_2_: peripheral measured hemoglobin oxygen saturation; OPIE: Oklahoma Premorbid Intelligence Estimation method

Table 1 Characteristics of the studies

Table 2 Measures used to evaluate cognition in the included articles

| **Author** | **Cognitive test** |
| --- | --- |
| **Hopkins RO^22^, 1999** | WAIS-R, WMS-R, RAVLT, Rey-Osterrieth Complex Figure Test, Trail Making Test parts A and B, Verbal fluency test |
| **Rothenhäusler H-B^13^, 2001** | SKT |
| **Hopkins RO^23^, 2004** | WAIS-R, WMS-R, RAVLT, Rey-Osterrieth Complex Figure Test, Trail Making Test parts A and B, Verbal fluency test |
| **Hopkins RO^24^, 2005** | WAIS-R, WMS-R, RAVLT, Rey-Osterrieth Complex Figure Test, Trail Making Test parts A and B, Verbal fluency test |
| **Risnes I^12^, 2006** | WAIS-R, Grooved Pegboard, Digit symbol, Trail Making Test parts A and B, RAVLT, Rey complex figure test, control oral word association, digit span, Stroop color word interference test |
| **Mikkelsen ME^10^, 2012** | Validated telephone battery |
| **Holzgraefe B^25^, 2016** | WAIS-IV (FSIQ and GAI), RAVLT, Rey-Osterrieth Complex Figure Test, WMS-III |

WAIS-R: Wechsler Adult Intelligence Scale-Revised; WMS-R: Wechsler Memory Scale-Revised; SKT: short cognitive performance test for assessing memory and attention; RAVLT: Rey Auditory Verbal Learning Test; FSIQ: Full Scale Intelligence Quotient; GAI: General Ability Index; WAIS-IV: Wechsler Adult Intelligence Scale, fourth edition; WMS-III: Wechsler Memory Scale, third edition

Table 3 Variable definition and results of the described investigations

| Author | Definition of hypoxaemia | Mean time of hypoxaemia  (hours) | Definition of cognitive impairment | Number and percentage of patients with cognitive impairment | Correlation of hypoxemia and cognitive impairment | Correlation of ECMO and cognitive impairment | Statistics |
| --- | --- | --- | --- | --- | --- | --- | --- |
| Hopkins RO^22^, 1999 | SaO_2_ <85% | <90% = 122 ± 144 hours  <85% = 13 ± 28 hours  <80% = 1 ± 3 hours | Comparison with corrected t-scores from normative population | 55 at dc (100%) 30% 17 of 55 after 1 year in WAIS-R (30%)  43 of 55 in WMS-R and RAVLT (78%) | PaO2 at enrollment was significantly related to cognitive outcome  FSIQ impairment for SaO2<90%, p=0.00, SaO2 <85%, p=0.008, SaO2<80%, p=0.013 at 1 yr follow up | - | ANOVA, paired t-test and Pearson correlations |
| Rothenhäusler H-B^13^, 2001 | not defined | not defined | SKT score of IQ | 11 of 46 (23.9%) | not tested | Not significant, p=0.330  numbers not shown | Mann-Whitney U test, Wilcoxon signed rank test, Kruskal- Wallis test, Spearman correlation |
| Hopkins RO^23^, 2004 | SaO_2_ <90% | SaO_2_ 105.9 hours ± 127.6 hours <90% | Two or more tests > 1.5 SD below normative population mean or 1 test below 2 SD below | 46 of 66 at dc (69.9%)  30 of 66 at one year (45%) | Not significant | - | McNemar |
| Hopkins RO^24^, 2005 | SaO_2_<90% | <90% = 106 ± 128 hours | 2 or more test deviation greater than 1.5 SD or greater 2SD in one test | Patients who completed 1 year follow up:  46 of 66 at dc (70%)  30 of 66 at 1 year (46%)  Patients who completed 2 year follow up:  29 of 62 (47%) | At discharge but not significant at 1 and 2 years of follow up, no p value shown | - | Descriptive, RMANOVA, Pearson correlation |
| Risnes I^12^, 2006 | not defined | not defined | 1 SD below normative population mean | 16 of 28 (57%).  8 of 16 with v-a ECMO (50%)  4 of 12 with v-v ECMO (33%) | not tested | Not significant, numbers not shown | Wilcoxon signed rank test |
| Mikkelsen ME^10^, 2012 | Not defined | unclear | Hayling Sentence Completion Test Score | 41 of 75 (55%) | Lower PaO_2_ was significantly associated with cognitive impairment at 12 months (p=0.02). PaO2 was no longer significantly associated in secondary analysis (p=0.32) | - | Multivariable logistic regression |
| Holzgraefe B^25^ 2016 | SaO_2_≤94% | 10 days or whole treatment if shorter than 10 days | 2 SD below normative population mean | 0 | none | no | no statistics |

BGA: blood gas analysis; SaO_2_: hemoglobin oxygen saturation; IQ: intelligence quotient; v-a ECMO: veno-arterial ECMO; v-v ECMO: veno-venous ECMO; dc: discharge; SKT: short cognitive performance test for assessing memory and attention; WAIS-R: Wechsler Adult Intelligence Scale-Revised; WMS-R: Wechsler Memory Scale-Revised; RAVLT: Rey Auditory Verbal Learning Test; FSIQ: Full Scale Intelligence Quotient
